# Supplementary material for: FERN – a Java framework for stochastic simulation and evaluation of reaction networks
Source: BMC Bioinformatics. 2008 Aug 29;9:356. doi: 10.1186/1471-2105-9-356 (PMC2553347; doi:10.1186/1471-2105-9-356)
Supplement: Additional file 1 — FERN distribution, Version 1.3. This archive contains the FERN source code and binaries as well as documentation and example models in FernML and SBML. [file 1471-2105-9-356-S1.zip › fern/doc/javadoc/fern/tools/functions/class-use/Probability.html]

Uses of Interface fern.tools.functions.Probability


---


|  |  |  |
| --- | --- | --- |
| **Overview** \| **Package** \| **Class** \| **Use** \| **Tree** \| **Deprecated** \| **Index** \| **Help** | |  |
| PREV   NEXT | **FRAMES**    **NO FRAMES**     **All Classes** |  |


---


## **Uses of Interface fern.tools.functions.Probability**

| Packages that use Probability | |
| --- | --- |
| **fern.network.creation** | Provides classes for the evolution of networks. |
| **fern.tools.functions** | Provides common function delegates and implementations. |

Uses of Probability in fern.network.creation

Constructors in fern.network.creation with parameters of type Probability

`AutocatalyticNetwork(char[] monomers,
Probability createProb,
Probability catProb,
int maxLength)`
  
          Creates the autocatalytic network from given monomers, reaction probability, catalysis probability up to a
given polymer length.

`AutocatalyticNetwork(char[] monomers,
Probability createProb,
Probability catProb,
int maxLength,
boolean useFastMethod)`
  
          Creates the autocatalytic network from given monomers, reaction probability, catalysis probability up to a
given polymer length. useDefault should usually set to true unless you want to evolve really huge networks.

Uses of Probability in fern.tools.functions

| Classes in fern.tools.functions that implement Probability | |
| --- | --- |
| `static class` | `Probability.Constant`             Implementation that yields a constant (and hence independent) probability. |
| `static class` | `Probability.ReactionProbability`             Implementation for the reaction probability for the autocatalytic network creation. |

---


|  |  |  |
| --- | --- | --- |
| **Overview** \| **Package** \| **Class** \| **Use** \| **Tree** \| **Deprecated** \| **Index** \| **Help** | |  |
| PREV   NEXT | **FRAMES**    **NO FRAMES**     **All Classes** |  |


---
